# Monoclonal antibodies to Cache Valley virus for serological diagnosis

**DOI:** 10.1371/journal.pntd.0010156

**Published:** 2022-01-24

**Authors:** Benjamin Skinner, Sierra Mikula, Brent S. Davis, Jordan A. Powers, Holly R. Hughes, Amanda E. Calvert

**Affiliations:** Arboviral Diseases Branch, Division of Vector-Borne Diseases, Centers for Disease Control and Prevention, Fort Collins, Colorado, United States of America; Liverpool School of Tropical Medicine, UNITED KINGDOM

## Abstract

Cache Valley virus (CVV) is a mosquito-borne virus in the genus *Orthobunyavirus*, family *Peribunyaviridae*. It was first isolated from a *Culiseta inorata* mosquito in Cache Valley, Utah in 1956 and is known to circulate widely in the Americas. While only a handful of human cases have been reported since its discovery, it is the causative agent of fetal death and severe malformations in livestock. CVV has recently emerged as a potential viral pathogen causing severe disease in humans. Currently, the only serological assay available for diagnostic testing is plaque reduction neutralization test which takes several days to perform and requires biocontainment. To expand diagnostic capacity to detect CVV infections by immunoassays, 12 hybridoma clones secreting anti-CVV murine monoclonal antibodies (MAbs) were developed. All MAbs developed were found to be non-neutralizing and specific to the nucleoprotein of CVV. Cross-reactivity experiments with related orthobunyaviruses revealed several of the MAbs reacted with Tensaw, Fort Sherman, Tlacotalpan, Maguari, Playas, and Potosi viruses. Our data shows that MAbs CVV14, CVV15, CVV17, and CVV18 have high specific reactivity as a detector in an IgM antibody capture test with human sera.

## Author summary

Cache Valley virus is a mosquito-borne virus found throughout the Americas. It causes fetal death and severe malformations in livestock, and only a few cases of human viral disease have been identified. Currently, we do not fully understand the spectrum of disease in humans including its potential to cause fetal malformations. The only serological diagnostic assay available to detect recent viral infection is plaque reduction neutralization test which requires the use of live virus in biocontainment. In order to develop faster and safer serodiagnostics we generated 12 monoclonal antibodies for incorporation into new assays. These antibodies are specific to the nucleoprotein of the virus and cross-react with other closely related mosquito-borne viruses. Four of these antibodies were incorporated into an immunoassay for the detection of IgM from human sera demonstrating their utility in serodiagnosis. Rapid and higher throughput assays utilizing these antibodies will expand diagnostic capacity and facilitate research to increase our understanding of Cache Valley disease prevalence and the virus’s impact on at-risk populations.

## Introduction

Cache Valley virus (CVV) is a member of the Bunyamwera serogroup in the genus *Orthobunyavirus*, family *Peribunyaviridae*. Subtypes of CVV include Tlacotalpan and Cholul viruses found in Mexico. Other viruses in the Bunyamwera serogroup of medical importance related to CVV include Maguari and Fort Sherman viruses [1–3]. CVV was first isolated from a *Culiseta inorata* mosquito in Cache Valley, Utah in 1956 [4]. Since then, it has been isolated from several mosquito species and mammals throughout the Americas and is considered the most widely distributed member of the Bunyamwera serogroup [1,2,5].

Orthobunyaviruses have a tripartite single stranded RNA genome encoding three structural proteins. The nucleocapsid (N) protein is approximately 23–27 kilodalton (kDa) in size and encapsidates the segmented genome together with several copies of the viral RNA-dependent RNA polymerase. The nucleocapsid core is surrounded by a lipid bilayer with viral glycoproteins, Gc (108–125 kDa) and Gn (29–41 kDa), displayed on the surface of the virion. These glycoproteins are involved in receptor-mediated endocytosis and viral-cell membrane fusion [6].

While the virus transmission cycle is not well characterized, serosurveillance studies have shown exposure to CVV occurring in domestic animals including sheep, horses, and cattle across North America [7,8]. Anti-CVV neutralizing antibody (Ab) in sheep was found to be as high as 28% and 64.6% in the U.S. and Saskatchewan, Canada, respectively [7,8]. CVV infection in sheep has been shown to be the causative agent of congenital malformations and fetal death [9]. Several studies demonstrate ovine CVV infection in utero compromises the musculoskeletal and central nervous systems [9–12].

The evidence of CVV infection incidence in humans is documented in serosurveys conducted throughout North and Central America with seroprevalence recorded as low as 3% and as high as 18% among populations sampled [13–15]. Less than 10 human cases of severe Cache Valley disease have been documented. At least five of these cases caused severe neurological symptoms including meningitis and encephalitis. Often in cases of human CVV infection, a positive diagnosis was the result of virus isolation from cerebral spinal fluid or blood [3,16–20]. While the teratogenicity of CVV in humans is unknown, serologic studies conducted retrospectively from specimens collected from women post-partum (n = 500) in the early 1960s showed anti-CVV Abs were significantly higher in those women who had given birth to newborns with macrocephaly [21].

While CVV exposure may be more common than clinical disease, this discrepancy may in fact be due to the lack of diagnostic testing conducted. Currently, no commercial diagnostic test or kit is available for the detection of CVV. Molecular assays such as reverse transcriptase polymerase chain reaction (RT-PCR) have been developed, but because of the potential for transient viremia in orthobunyaviral infections, cases of CVV may be missed [22]. Serological techniques for diagnosis are limited to plaque reduction neutralization test (PRNT) which requires a dedicated biosafety laboratory for work with live virus, and positive titers in one sample cannot indicate the timing of infection. Rapid immunoassays, such as enzyme-linked immunosorbent assay (ELISA) or multiplex immunoassay (MIA) for faster serological detection of IgM Abs present early in infection, are needed to overcome the limitations of PRNT. Additionally, immunoassays for the detection of IgG Abs can aid in identification of past CVV infections for increased serosurveillance. The limitation in these assays is that they all require monoclonal Abs (MAbs) in their design. IgM antibody capture ELISA (MAC-ELISA) requires a virus-specific MAb-enzyme conjugate as a detector, while IgG ELISA uses a virus-specific MAb to capture and present antigen for the detection of anti-viral IgG.

To our knowledge, no MAbs reactive to CVV have been described. Therefore, our objective was to develop anti-CVV MAbs for inclusion in diagnostic immunoassays for the detection of both recent and prior infections and for use in future studies to assess the disease burden of CVV in the United States. Here, we report the development and characterization of 12 murine MAbs for future application. All MAbs developed were non-neutralizing and reactive with CVV in ELISA formats. Several of these MAbs were cross-reactive with related orthobunyaviruses including Tensaw, Fort Sherman, Tlacotalpan, Maguari, Playas, and Potosi viruses. In preliminary experiments, four MAbs showed their potential to function as a detector in a MAC-ELISA format for detection of anti-CVV IgM from human sera.

## Materials and methods

### Ethics statement

Ethics approval for use of previously collected human diagnostic specimens was obtained from CDC’s Human Subjects Institutional Review Board (CDC IRB# 6773). All specimens were delinked from any personal identifiers prior to the commencement of this study.

Mice were handled as specified by the Division of Vector-Borne Diseases Institutional Animal Care and Use Committee recommendations (Protocol #19–003).

### Viruses, cell lines, and diagnostic specimens

CVV strain W1-03BS7669 (genotype 1), Tensaw virus (TENV) strain A9-171B, Tlacotalpan virus (TLAV) strain 61D240, Fort Sherman virus (FSV) strain 86MSP18, Maguari virus (MAGV) strain BeAr7272, Playas virus (PLAV) strain 75V5938, Potosi virus (POTV) strain IL94-1921, and La Crosse virus (LACV) strain Original were obtained from the Arboviral Diseases Branch (ADB), Reference and Reagent Laboratory (Fort Collins, CO, USA). CVV stock was grown in Vero cells to a titer of 5.0 × 10^7^ plaque forming units per mL (PFU/mL). Vero cells were grown in DMEM (Cat# 11965118, Invitrogen, Waltham, MA, USA) supplemented with 10% fetal bovine serum (FBS) (Cat# 1500-500G, VWR, Radnor, PA, USA), 2 mM L-glutamine (Cat# 25030–081 Invitrogen, Waltham, MA, USA), 0.15% sodium bicarbonate (Cat# 25080–094, Gibco, Waltham, MA, USA), 100 U/mL penicillin G sodium, and 100 μg/mL streptomycin (Cat # 15140–122, Gibco, Waltham, MA, USA) at 37°C in 5% CO_2_. CVV was purified on glycerol tartrate gradients and protein concentration was determined by Bradford assay (Cat# 5000006, Bio-Rad, Hercules, California, USA) as previously described [23]. These purified stocks were used in subsequent serologic assays. The mouse myeloma cell line, P3X63Ag8.653, used for hybridoma fusions was grown in ClonaCell-HY Medium A (Cat# 03801, StemCell Technologies, Vancouver, British Columbia, Canada) at 37°C in 5% CO_2_.

One human serum sample positive for CVV (confirmed by PRNT with 90% endpoint cut off (PRNT_90_) with an endpoint titer of 1:1280) and eight other human serum samples positive for other arboviral diseases with no indication of a recent CVV infection were obtained from the ADB’s Diagnostic lab for evaluation of MAbs’ performance in the MAC-ELISA format. Pooled negative human sera (NHS) (Cat# S1-100ML, EMD Millipore, Burlington, MA, USA) was used as a negative control and confirmed to have no reactivity to several arboviruses, including CVV, by PRNT_90_.

### Animal studies

AG129 mice deficient in interferon (IFN) α/β and γ receptors were originally obtained from B&K Universal (Hull, United Kingdom) and bred in-house. Virulence of CVV in 6-week-old AG129 mice was evaluated by inoculating five groups of mice (n = 6) intraperitoneally (IP) with 10-fold dilutions of virus (10^3^ to 10^−1^ PFU/0.1 mL). After challenge, mice were monitored four times per day for signs of morbidity (hunched posture, ruffled fur, and neurological signs) for four weeks. Mice that showed signs of morbidity were euthanized immediately. Fifty percent IP lethal dose endpoints (IP LD_50_) were calculated as previously described [24]. The first immunization of surviving mice (n = 4) inoculated in the 10 PFU group were immunized with 25 μg β-propiolactone inactivated CVV and Magic Mouse adjuvant (Cat# CDN-A001, Creative Diagnostics, Shirley, NY, USA). This immunization occurred 38 days after viral challenge and was considered day 0 in the immunization timeline. The final boost before hybridoma fusions was performed 81 days after the first immunization. Sera was collected 31 days post-viral challenge and 35-, 66-, and 85- days post-immunization (PI) from the first inoculation of inactivated virus and adjuvant.

### Hybridoma fusions, clone selection, and MAb purification

At day of harvest, mice were anesthetized under 1–4% isoflurane by inhalation, and euthanasia was carried out by cervical dislocation. Splenocytes from vaccinated mice were harvested four days after the last inoculation. Electrofusions with the mouse myeloma cell line P3X63Ag8.653 were performed with the ECM2001 electrofusion system (BTX, Holliston, MA, USA) in glass microslides with a 10 mm gap according to the manufacturer’s protocol. After 48 hours of recovery, fused cells were plated in ClonaCell-HY hybridoma semi-solid selection medium (Cat# 03804, StemCell Technologies, Vancouver, British Columbia, Canada) containing anti-mouse IgG (H+L) CloneDetect (Cat# K8220, Molecular Devices, Sunnyvale, CA, USA) and incubated for 10 days at 37°C in 5% CO_2_. Clones expressing anti-mouse IgG were identified and picked with the ClonePix2 automated colony picker (Molecular Devices, Sunnyvale, CA, USA) according to the manufacturer’s protocol. Clones were grown in 96-well plates in ClonaCell-HY hybridoma medium containing hypoxanthine and thymidine (Cat# 03805, StemCell Technologies, Vancouver, British Columbia, Canada). Positive clones were selected after screening hybridoma supernatant by ELISA and expanded in CD hybridoma medium (Cat#11279023, ThermoFisher Scientific, Waltham, MA, USA) supplemented with 2% low IgG FBS (Cat# 16250078, ThermoFisher Scientific, Waltham, MA, USA). Up to 500 mL of hybridoma supernatant was concentrated using centrifugal concentrators (Cat# UFC710008, Millipore Sigma, Burlington, MA, USA) with a 100 kDa cut off and dialyzed overnight in 0.1M phosphate buffer. MAbs were purified using protein-A Sepharose (Cat# 101041, GE Healthcare, Pittsburgh, PA, USA) according to the manufacturer’s instructions. MAb concentrations were determined using a NanoDrop ND1000 spectrophotometer (ThermoFisher Scientific, Waltham, MA, USA).

### Isotyping assay

Ab isotypes were determined using the Antibody Isotyping 7-Plex Mouse ProcartaPlex kit (Cat# EPX070-20815-901, ThermoFisher Scientific, Waltham, MA, USA) according to the manufacturer’s protocol.

### Enzyme linked-immunosorbent assay (ELISA)

All ELISAs were performed in 96-well plates (Cat# 44-2404-21, Nunc Maxisorp plates, ThermoFisher Scientific, Waltham, MA, USA). Plates were coated either with 0.25 μg/well of purified CVV or anti-CVV rabbit polyclonal Ab (Lot# A43977, available upon request from ADB, Reference and Reagent Laboratory, Fort Collins, CO, USA) diluted 1:500 in carbonate/bicarbonate buffer (50 mM sodium carbonate, 50 mM sodium bicarbonate, pH 9.6) and incubated overnight at 4°C. Plates were washed five times with PBS/0.1% Tween wash buffer with an automatic plate washer. Nonspecific binding sites were blocked with Pierce Starting Block (PBS) blocking buffer (Cat# 37538, ThermoFisher Scientific, Waltham, MA, USA) (100 μL/well) and immediately removed. To plates coated with anti-CVV rabbit polyclonal sera, 0.25 μg/well of purified CVV was added (50 μL/well) and incubated on the plates for two hours at 37°C, after which the plates were washed as previously described. Purified MAbs starting at 10^4^ ng/mL or mouse sera starting at a 1:100 dilution in PBS were added in 4-fold dilutions (50 μL/well) and incubated for one hour at 37°C. Plates were washed five times before the addition of goat anti-mouse Ab conjugated to horseradish peroxidase (Cat# 115-035-062, Jackson ImmunoResearch, West Grove, PA, USA) (50 μL/well), diluted 1:5000 in 5% skim milk/PBS. After an incubation period of one hour at 37°C, plates were washed again 10 times. Enhanced K-blue TMB substrate (Cat# 308175, Neogen, Lansing, MI, USA) was added to each well of the plate (100 μL/well) and incubated in the dark at room temperature for 10 minutes. The reaction was stopped with the addition of 2N H_2_SO_4_ (50 μL/well), and the plates were read at 450 nm. Binding curves were generated by a 4-parameter non-linear regression dose response with the bottom of the curve constrained to 0 and used to calculate the half-maximal effective concentration (EC_50_) values of each MAb using GraphPad Prism V6.

### Immunoblot

CVV proteins from a purified virus preparation were separated by sodium dodecyl sulphate-polyacrylamide gel electrophoresis (SDS-PAGE) on 4–12% Bis-Tris gels (Cat# NP0335PK2, ThermoFisher Scientific, Waltham, MA, USA) under reduced (DTT (Cat# NP0004, ThermoFisher Scientific, Waltham, MA, USA)) and non-reduced conditions. Proteins were electrophoretically blotted from the gels onto nitrocellulose membranes (Cat# 88014, ThermoFisher Scientific, Waltham, MA, USA) and washed for 15 minutes in PBS/0.1% Tween wash buffer. Nonspecific binding sites were blocked with 3% bovine serum albumin (BSA) in PBS overnight at 4°C. Sera taken from vaccinated mice (diluted 1:100 in PBS) and purified MAbs (50 μg/mL) were incubated with the membrane for one hour with gentle rocking. Membranes were washed again in PBS/0.1% Tween wash buffer three times for five minutes each. Goat anti-mouse Ab conjugated to alkaline phosphatase (Cat# 115-055-062, Jackson ImmunoResearch, West Grove, PA, USA) was diluted 1:200 in 3% BSA/PBS and incubated on the membrane for one hour with gentle rocking. Membranes were washed as described previously, and BCIP/NBT phosphatase substrate (Cat# 5420–0038, SeraCare, Milford, MA, USA) was added to the membrane until a color change appeared. The reaction was stopped by the addition of water. CVV strain 6V633 mouse-hyperimmune ascitic fluid (MHIAF) (lot# M3233, available upon request from ADB, Reference and Reagent Laboratory, Fort Collins, CO, USA) was used as a positive control to detect all viral proteins while PBS alone was used as a negative control to detect nonspecific binding of the Ab conjugate to viral protein.

### Plaque reduction neutralization test (PRNT)

To assess neutralization activity, purified MAbs and mouse sera were tested in duplicate in PRNT with CVV. About 100 PFU of CVV strain W1-03BS7669 were incubated with equal amounts of serial 2-fold dilutions of purified MAb starting at 10^4^ ng/mL for one hour at 37°C. Sera were diluted 1:5 in BA1 (Hanks M-199 salts (Cat# M9163, Sigma-Aldrich, St. Louis, MO, USA), 0.05 M Tris pH 7.6 (Cat# 15567027, Gibco, Waltham, MA, USA), 1% bovine serum albumin (Cat# 81-066-4, Millipore, Burlington, MA, USA), 0.35 g/L sodium bicarbonate (Cat# 25080–094, Gibco, Waltham, MA, USA), 100 U/mL penicillin, 100 mg/L streptomycin (Cat # 15140–122, Gibco, Waltham, MA, USA), 1 mg/L fungizone (Cat# SV30078.01, Hyclone, Marlborough, MA, USA), and heat inactivated at 56°C for 30 minutes before mixing with virus as described above. Six-well plates of Vero cells were then inoculated with the virus–Ab mixtures and incubated at 37°C with 5% CO_2_ for one hour after which cells were overlaid with 3 mL of medium containing 1% SeaKem LE agarose (Cat# 50004, Lonza, Rockland, ME, USA) in nutrient medium (0.165% lactalbumin hydrolysate (Cat# 259962, VWR, Radnor, PA, USA), 0.033% yeast extract (Cat#212750, ThermoFisher Scientific, Waltham, MA, USA), Earle’s balanced salt solution, and 2% FBS). After incubation at 37°C for two days a second overlay containing an additional 80 μg/mL of neutral red vital stain (Cat# 091691149, MP Biomedicals, Santa Ana, USA) was added. Plaques were counted three days after infection and percent neutralization was calculated based on input virus titer. Virus neutralization curves were generated by a 4-parameter non-linear regression dose response used to calculate the half-maximal inhibitory concentration (IC_50_) values of each MAb using GraphPad Prism V6 with the bottom and top of the curves constrained to 0 and 100, respectively.

### Immunofluorescence assay (IFA)

To determine the cross-reactivity of anti-CVV MAbs with other orthobunyaviruses, Vero cells grown on coverslip slides were infected at a multiplicity of infection (MOI) of 1.0 with viruses mentioned previously, and cells were fixed 24 hours later with cold 70% acetone in PBS for 10 minutes. Purified CVV MAbs diluted to 10 μg/mL were added (20 μL/well) and incubated at 37°C for 30 minutes. MHIAFs generated against each orthobunyavirus were diluted 1:500 in PBS (CVV MHIAF (Lot# M32331-13), TENV MHIAF (Lot# A4862-1Aby), TLAV MHIAF (Lot# M8158A/Aby), FSV MHIAF (Lot# M27862Aby), MAGV MHIAF (Lot# PR102130-Aby), PLAV MHIAF (Lot# M15472-1Aby), POTV MHIAF (Lot# M25656A/Aby), and LACV MHIAF (Lot# M30177Aby) available upon request from ADB, Reference and Reagent Laboratory, Fort Collins, CO, USA) and used as a positive control on cells infected with the homologous virus (S2 Fig). A purified anti-alphavirus IgG2b MAb was used as a negative Ab control (NAC) to detect nonspecific binding of the Ab conjugate to virus-infected cells (S2 Fig). Slides were washed three times in PBS and allowed to dry before goat anti-mouse conjugated to fluorescein isothiocyanate (FITC) (Cat# 115-095-062, Jackson ImmunoResearch, West Grove, PA, USA) diluted 1:100 in PBS was added (20 μL/well) and incubated at 37°C for 30 minutes. As another control, uninfected cells grown on coverslips were fixed and stained with each MAb and control Ab as previously described (S2 Fig). Slides were washed and mounted with ProLong Gold antifade reagent with DAPI (Cat# P36931, ThermoFisher Scientific, Waltham, MA, USA) and examined on a LSM 800 laser scanning confocal microscope (Carl Zeiss, Oberkochen, Germany).

### IgM-antibody capture pilot ELISA (MAC-ELISA)

The MAbs with the highest reactivities in the indirect and capture ELISAs were tested for their preliminary performance as detector Abs in the MAC-ELISA format using one CVV positive human sera, eight human sera diagnosed with a recent infection of an unrelated arbovirus, and pooled negative human sera (NHS) used as a negative control. Goat anti-human IgM Ab (Cat# 5210–0157, SeraCare, Milford, MA, USA) was diluted 1:2000 in carbonate/bicarbonate buffer and incubated overnight at 4°C. Plates were washed five times with PBS/0.1% Tween wash buffer with an automatic plate washer. Nonspecific binding sites were blocked with 5% nonfat dry milk with 0.5% Tween-20 in PBS (100 μL/well) for 30 minutes at room temperature, after which plates were washed. Human serum diluted 1:400 in PBS was added to the plates (50 μL/well) in triplicate and incubated at 37°C for one hour. Plates were washed before the addition of positive antigen (purified CVV 0.25 μg/well diluted in PBS) or negative antigen (PBS alone) and incubated overnight at 4°C. The next day, plates were washed, purified MAb diluted to 1 μg/mL in PBS was added to the plate (50 μL/well), and the plate was incubated for one hour at 37°C. Plates were washed five times before the addition of goat anti-mouse Ab conjugated to horseradish peroxidase (Cat# 115-035-062, Jackson ImmunoResearch, West Grove, PA, USA) (50 μL/well), diluted 1:5000 in 5% skim milk/PBS. After an incubation period of one hour at 37°C, plates were washed again 10 times. Enhanced K-blue TMB substrate (Cat# 308175, Neogen, Lansing, MI, USA) was added to each well of the plate (100 μL/well) and incubated in the dark at room temperature for 10 minutes. The reaction was stopped with the addition of 2N H_2_SO_4_ (50 μL/well), and the plates were read at 450 nm. Nonspecific background values (NBVs) were determined by calculating positive/negative (P/N) ratios of the serum sample on viral antigen and negative antigen wells (average optical density (OD) of the serum sample on positive antigen divided by the average OD of the serum sample on negative antigen). Pilot diagnostic P/N ratios were calculated by taking the OD values of the test serum sample on viral antigen and dividing by the OD values of NHS on viral antigen.

## Results

### Virulence and immunogenicity of CVV in AG129 mice

**Fig 1:**
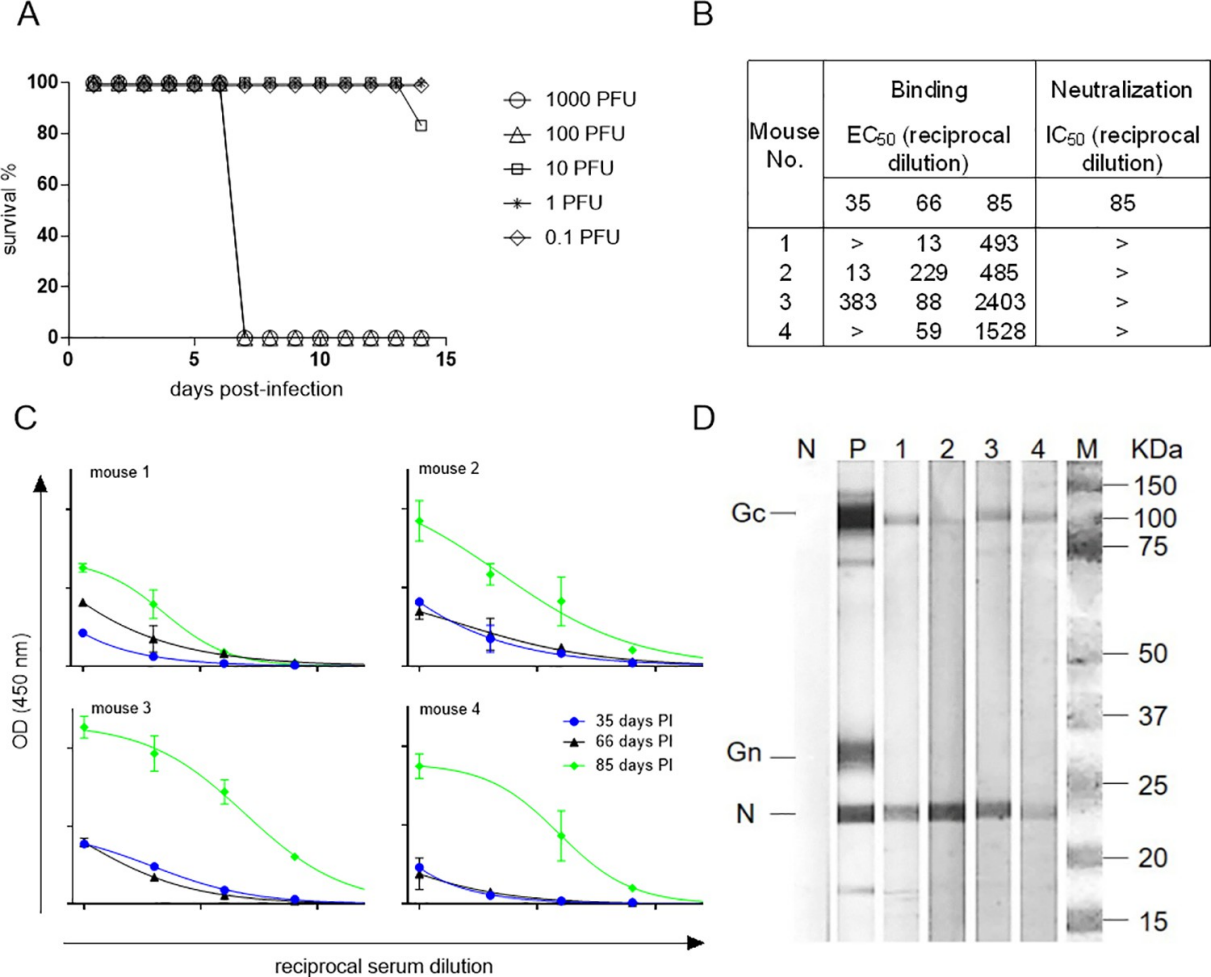
Virulence of CVV and anti-CVV specific Ab response in AG129 mice. A) Mice (n = 6/group) were inoculated IP as described with 10-fold dilutions of CVV and monitored for signs of morbidity. 1000 PFU (open circle), 100 PFU (open triangle), 10 PFU (open square), 1 PFU (star), and 0.1 PFU (open diamond). B) and C) Binding of anti-CVV Ab from immunized mice to purified virus was determined by indirect ELISA with 4-fold dilutions of sera. Colors indicate day PI (day 35 (blue), day 66 (black), day 85 (green)). EC_50_ values, shown as the reciprocal of dilution of sera, were determined by non-linear fit analysis, with the bottom of the curve constrained to 0, using GraphPad Prism software V6. EC_50_ values were calculated for 35-, 66- and 85-days PI. IC_50_ values for 85-days PI were determined by non-linear fit analysis, with the bottom and top of the curve constrained to 0 and 100, respectively, using GraphPad Prim software V6. > indicates no detectable binding reactivity or neutralizing activity at a 1:10 dilution of sera. D) CVV protein-specific Ab responses in AG129 mice 85-days PI. Lanes: N (negative control (PBS)), P (positive control (CVV MHIAF)), 1 (mouse 1), 2 (mouse 2), 3 (mouse 3), 4 (mouse 4), M (Bio-Rad Precision Plus Protein Dual Color Standard (Cat# 1610374)), Gc (glycoprotein C), Gn (glycoprotein N), N (nucleoprotein), kDa (Kilodaltons).

To determine the virulence of CVV strain W1-03BS7669 in α/β and γ IFN-receptor-deficient AG129, 6-week-old mice were inoculated IP with 10-fold dilutions of virus starting at 10^3^ PFU/0.1 mL. The first signs of illness in mice infected with 10^3^ or 10^2^ PFU began on day 5 post-infection, and their condition rapidly deteriorated thereafter. Only one animal infected with 10^1^ PFU succumbed to infection 13 days post-infection. The IP LD_50_ of CVV in AG129 mice was calculated as 25 PFU/0.1 mL (Fig 1A). There was no Ab response measured by ELISA in surviving AG129 mice immunized with lower doses of CVV (10, 1.0, and 0.1 PFU/0.1 mL) indicating these animals may not have become infected or the level of Ab was below the limit of detection of the assay. Since no Ab response was detected, four of the surviving mice in the 10 PFU/0.1 mL group were subsequently immunized with purified inactivated CVV and adjuvant for hybridoma development. Two mice developed a detectable Ab response (EC_50_ reciprocal dilution values: 13 and 383 for mice 2 and 3, respectively) to CVV when tested 35-days PI, and all mice had detectable Ab to CVV 66 days after the first immunization (Fig 1B and 1C). At day of harvest (day 85 PI), mice 3 and 4 had the highest detectable Ab to CVV (EC_50_ reciprocal dilution values: 2403 and 1528, respectively), while mice 1 and 2 also displayed anti-CVV Ab measured by ELISA (EC_50_ reciprocal dilution values: 493 and 485, respectively) (Fig 1B and 1C). None of the mice produced neutralizing Ab as measured by PRNT_50_; however, sera from mouse 3 taken on day 85 PI did display 48.5% virus neutralization at a 1:10 dilution in the assay (Figs 1B and S1A). The Ab response developed in immunized mice was to viral structural proteins, Gc and N, as demonstrated by immunoblot under reduced conditions (Fig 1D).

### Generation and characterization of anti-CVV MAbs

**Fig 2:**
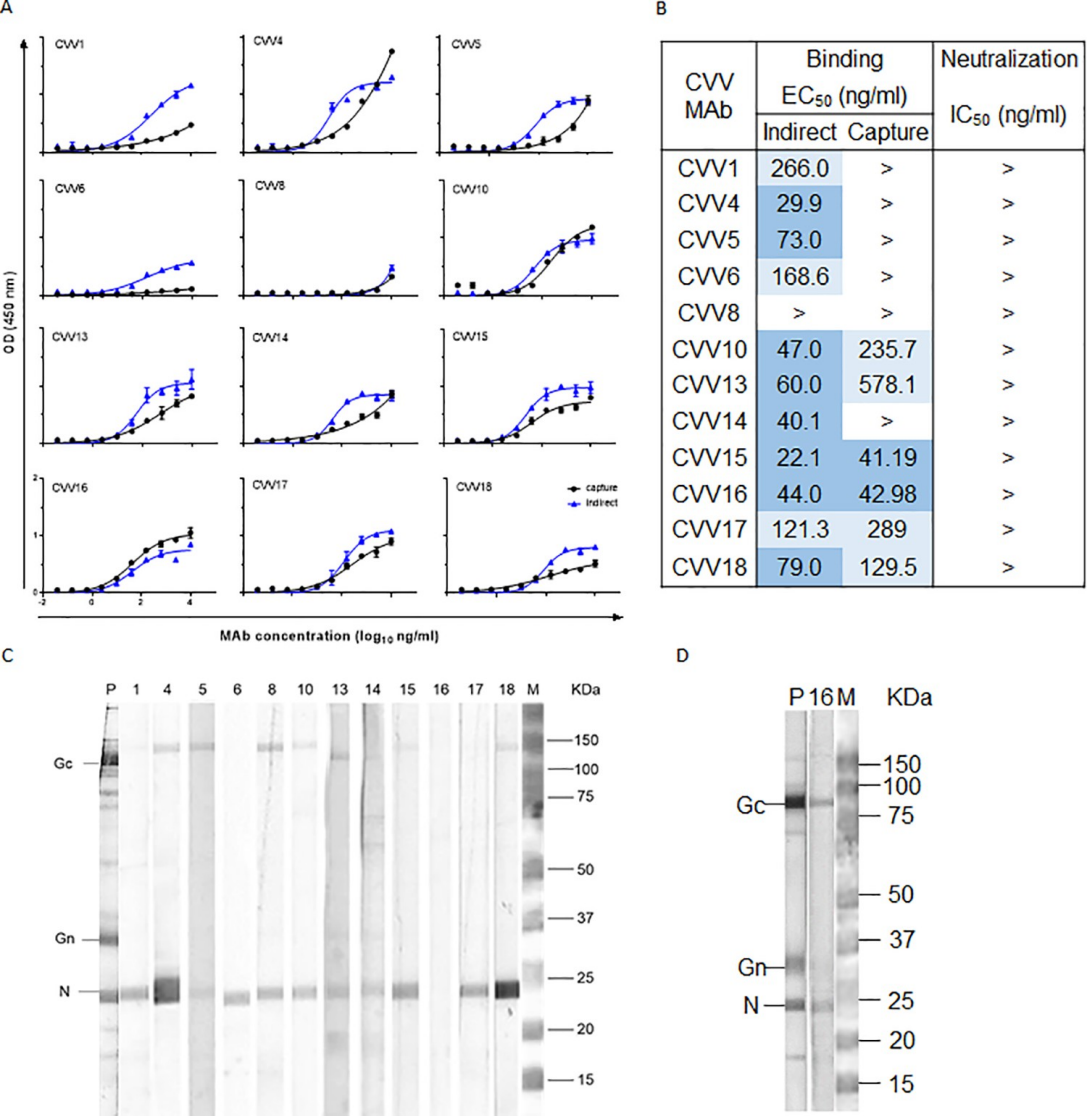
Binding, neutralization reactivity, and protein specificity of anti-CVV MAbs. A) Binding of anti-CVV MAbs to purified virus was determined by indirect (blue) and capture (black) ELISA. B) EC_50_ and IC_50_ values (shown as MAb concentrations of ng/mL) in ELISA and PRNT were determined by non-linear fit analysis, with the bottom of the curve constrained to 0 for both analyses and the top of the curve constrained to 100 for neutralization analysis, using GraphPad Prism software V6. Dark blue: EC_50_ values <100 ng/mL. Light blue: EC_50_ values 100–10,000 ng/mL. > indicates no detectable reactivity at 10,000 ng/mL. C) Western blot of reduced purified CVV with anti-CVV MAbs was prepared as described in Materials and Methods. Lane nomenclature corresponds to MAb nomenclature. D) Western blot of non-reduced purified CVV with MAb CVV16, P (positive control (CVV MHIAF)), M (Bio-Rad Precision Plus Protein Dual Color Standard (Cat# 1610374)), Gc (glycoprotein C), Gn (glycoprotein N) N (nucleoprotein), kDa (Kilodaltons).

**Fig 3:**
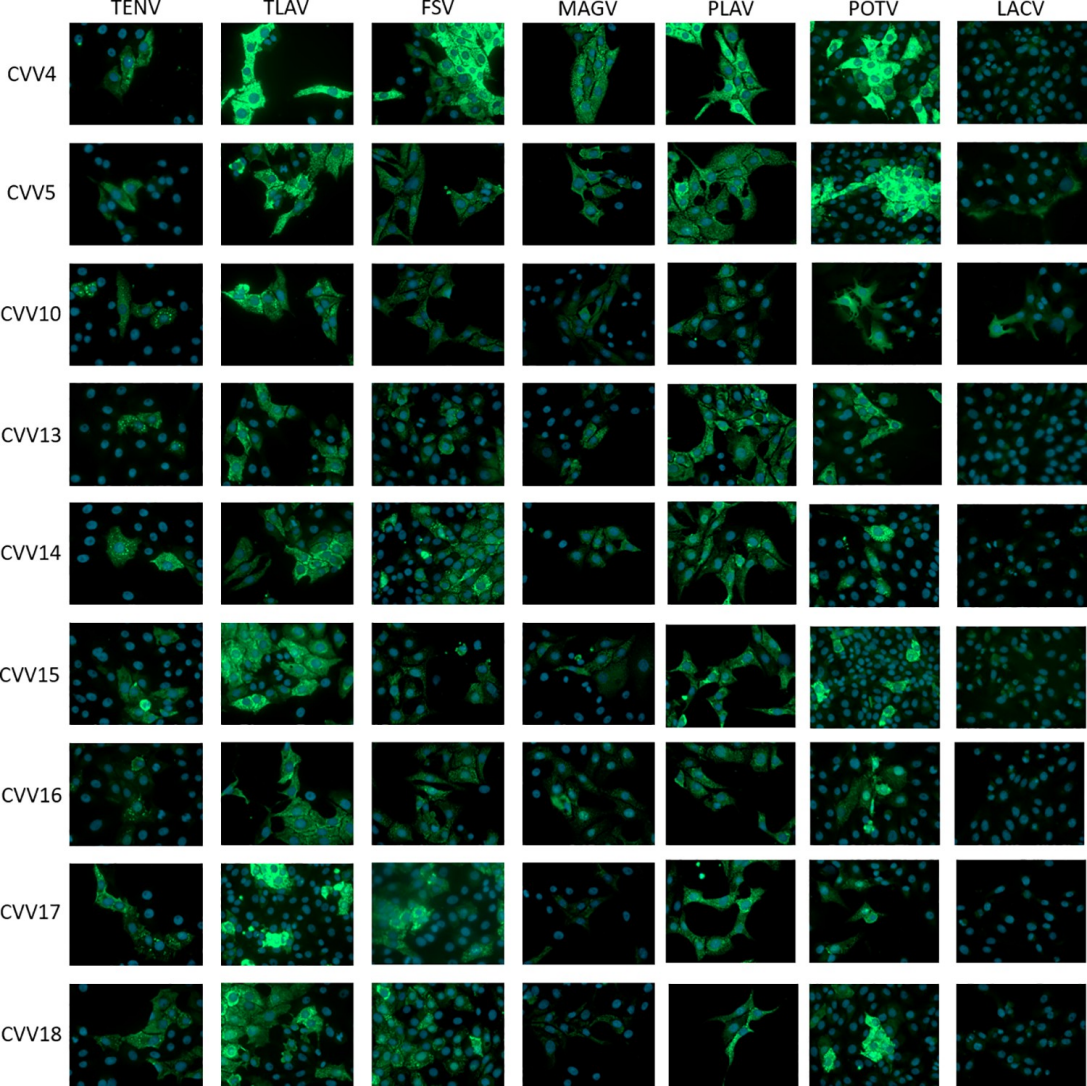
Cross-reactivity of anti-CVV MAbs with related orthobunyaviruses. Vero cells were grown on glass coverslips in 24-well plates, infected with viruses in the Bunyamwera serogroup at an MOI 1.0 and fixed 24 hours post-infection. Cells were stained with anti-CVV MAbs and a goat anti-mouse FITC labeled conjugate (green). Nuclei of cells were stained with DAPI (blue).

Thirty-five hybridoma clones secreting anti-CVV Ab were isolated, and of these hybridoma clones, 12 were further evaluated based on their ELISA reactivity in the selection process. All MAbs with detectable signal in the assay were determined to be IgG2b isotype (S1 Table). MAbs with EC_50_ values <100 ng/mL in the indirect ELISA were CVV4, CVV5, CVV10, CVV13, CVV14, CVV15, CVV16, and CVV18 (Fig 2A and 2B). MAbs CVV1, CVV6, and CVV17 had EC_50_ values between 100–10,000 ng/mL, while the EC_50_ value of CVV8 was >10,000 ng/mL in the indirect ELISA. The MAbs with EC_50_ values <100 ng/mL in the capture ELISA were CVV15 and CVV16 (Fig 2A and 2B). MAbs CVV10, CVV13, CVV17, and CVV18 had EC_50_ values between 100–10,000 ng/mL, while the EC_50_ values of CVV1, CVV4, CVV5, CVV6, CVV8, and CVV14 were >10,000 ng/mL. None of the MAbs were able to neutralize virus *in vitro* when tested by PRNT_50_ (Figs 2B and S1B). Viral protein specificity was determined by SDS-PAGE and Western blot. Most MAbs reacted specifically to the N protein of CVV under reduced conditions (Fig 2C). MAb CVV16, which showed no reactivity to viral proteins under reduced conditions, was reactive with Gc and N protein under non-reduced conditions (Fig 2D). Cross-reactivity with other closely related viruses was tested by immunofluorescence of virus-infected Vero cells. All MAbs exhibited some immunofluorescence signal with serologically related TENV, TLAV, FSV, MAGV, PLAV, and POTV when tested at a concentration of 10 μg/mL in IFA compared to the positive and negative controls (Figs 3 and S2). No or low signal intensities (CVV5 and CVV10) with CVV MAbs were noted on cells infected with LACV, a more distantly related orthobunyavirus.

### Development of pilot MAC-ELISA for CVV

Performances of nine anti-CVV MAbs with the lowest EC_50_ values in the indirect ELISA, as well as CVV17 that had detectable EC_50_ values in both the indirect and capture ELISAs, were assessed for their ability to be used as detector Abs in a pilot MAC-ELISA format using purified CVV as positive antigen. NBVs of each MAb were determined using the ratios of the absorbance of the serum samples on positive antigen over absorbance of the serum sample on negative antigen (P/N) tested in triplicate. Anti-CVV MAbs with the highest NBV P/N ratios with the CVV positive human serum sample were CVV14, CVV15, CVV17, and CVV18 with P/Ns of 12.9, 11.5, 14.5, and 12.2, respectively. NBV P/Ns of each MAb tested with the NHS remained <2.0 (Table 1). MAb CVV13 had a low NBV P/N (2.13) due to high nonspecific reactivity with the negative antigen control, while MAbs CVV4, CVV5, CVV10, and CVV16 had lower NBV P/Ns (6.94, 8.12, 6.39, and 9.22, respectively) compared to other MAbs tested (Table 1). MAbs CVV14, CVV15, CVV17, and CVV18 were further evaluated in the MAC-ELISA with diagnostic specimens confirmed positive for a recent infection with other arboviruses (Jamestown Canyon virus (JCV), LACV, Powassan virus (POWV), West Nile virus (WNV), and chikungunya virus (CHIKV)) with no indication of a recent CVV infection. P/N ratios in this assay were calculated as the absorbance of the diagnostic serum samples on positive antigen over absorbance of the NHS on positive antigen. P/N ratios for the CVV positive human serum were high with all MAbs used as detector Abs (Table 2). Some of the human serum samples (POWV, WNV, and CHIKV) had higher P/N ratios than other samples tested, although not as high as the CVV positive human serum. One of the serum samples diagnosed as a recent WNV infection had a P/N ratio >3.0 (a presumptive positive in other CDC MAC-ELISA assays) when MAb CVV14 was used as the detector Ab (Table 2). When MAb CVV18 was used as the detector Ab, two of the serum samples (POWV and WNV) had P/N ratios >3.0, and when MAbs CVV15 and CVV17 were used as the detector Abs, three of the serum samples (POWV, WNV, and CHIKV) had P/N ratios >3.0 (Table 2). These preliminary results demonstrate that MAbs CVV14, CVV15, CVV17, and CVV18 have the potential to be used as detector Abs in an optimized MAC-ELISA format for the detection of anti-CVV IgM Abs from human sera.

**Table 1:** Nonspecific background evaluation of anti-CVV MAbs as detector Abs in pilot MAC-ELISA.

MAb clone	NBV (P/N)^*a*^	
	CVV^*b*^	NHS^*c*^
CVV4	6.94	0.99
CVV5	8.12	0.93
CVV10	6.39	1.05
CVV13	2.13	0.89
CVV14	12.9	1.12
CVV15	11.5	1.01
CVV16	9.22	0.94
CVV17	14.5	1.18
CVV18	12.2	1.22

^*a*^NBV (nonspecific background values) were determined by calculating the ratio of the mean OD_450_ of the wells containing CVV (P) divided by the mean OD_450_ of the wells without virus (N) for each serum sample.

^*b*^human serum sample positive for CVV neutralizing Ab.

^*c*^pooled negative human serum (NHS).

**Table 2:** Specificity of pilot MAC-ELISA with anti-CVV MAbs as detector Abs.

MAb clone	P/N ratio^*a*^								
	CVV^*b*^	JCV	LACV	POWV	POWV	WNV	WNV	DENV	CHIKV
CVV14	27.52	1.40	1.48	1.40	1.45	4.01	1.45	1.10	2.68
CVV15	26.85	1.69	1.91	1.98	3.25	3.88	1.22	1.15	3.26
CVV17	59.66	1.51	2.09	2.49	4.83	5.43	1.79	1.17	3.49
CVV18	36.38	1.35	2.14	2.51	3.22	4.10	1.81	1.17	2.43

^*a*^Results are expressed as the P/N ratio of mean test patient serum OD_450_ values on CVV divided by mean negative patient serum OD_450_ values on CVV.

^*b*^Arbovirus diagnosis of patient serum confirmed by PRNT_90_. Jamestown Canyon virus (JCV), La Crosse virus (LACV), Powassan virus (POWV), West Nile virus (WNV), dengue virus (DENV) and chikungunya virus (CHIKV).

## Discussion

This study reports the generation of a panel of MAbs directed to CVV. Activated B cells from AG129 mice deficient in α/β and γ IFN receptors were infected and boosted with CVV for the development of these hybridomas since previous studies had shown immunocompetent mice are resistant to CVV infection [25]. While anti-CVV Ab was not detected in mice that survived challenge and whose splenocytes were subsequently used for hybridoma fusions, an undetectable Ab response post-infection may have resulted in increased Ab titers after boost with inactivated CVV.

The use of this mouse model for the development of MAbs to Heartland virus (HRTV), another arbovirus incapable of a productive viral infection in immunocompetent mice, has shown its utility in MAb development for use in a HRTV MIA for the rapid and accurate identification of recent and past human HRTV infections [26,27]. AG129 mice are also a valuable tool in studying the pathogenesis of arboviral disease due to their inability to produce sterilizing immunity in the absence of IFN, thus allowing for an unimpeded viral infection *in vivo*. Several IFN-induced immune proteins and regulators have been shown to be important in restricting growth *in vivo* for several viruses in the genuses *Orthobunyavirus* and *Phlebovirus* including CVV, LACV, Oropouche virus, and Rift Valley fever virus [11,28–33]. Recently, López et. al (2021) showed that IFN plays an important role in disease of CVV *in vivo*. Similar to our results, six-week-old IFN-αβR^-/-^ mice inoculated subcutaneously with 10^4^ PFU of CVV became moribund by eight days post-infection [34]. Our previous studies have shown that the lack of α/β and γ IFN receptors in AG129 mice and subsequent downregulated IFN responses results in susceptibility to a host of other arboviruses [27,35–38]. AG129 mice have also been used to investigate sexual transmission and teratogenicity of Zika virus, another arbovirus of public health concern [39]. Likewise, pregnant IFN-αβR^-/-^ mice have been used to study CVV vertical transmission resulting in spontaneous abortions and congenital malformations *in vivo* [34].

Bunyaviruses encode for three structural proteins: two glycoproteins (Gn and Gc) and nucleoprotein (N). The panel of anti-CVV MAbs were all specific to the viral N protein. The N protein is highly conserved within the Bunyamwera serogroup with many species sharing >90% amino acid sequence identity. The N protein is abundantly expressed in virus-infected cells; however, anti-N protein MAbs do not typically have neutralizing activity [40–42]. In contrast, most neutralizing MAbs are directed against the glycoproteins, particularly Gc [42–45]. The anti-CVV MAbs reactive to the N protein described here, cross-reacted with several viruses in the Bunyamwera serogroup by IFA including TLAV, FSV, MAGV, PLAV, and POTV; however, anti-CVV MAbs had little reactivity with the more distantly related LACV, another orthobunyavirus in the California serogroup. MAb CVV4 displayed the highest signal intensities to TLAV, FSV, PLAV, and POTV. Like CVV, these viruses commonly circulate in the Americas; however, they rarely cause human disease [46–48]. Anti-CVV MAbs developed here with cross-reactivity to closely related viruses may be useful in future studies to determine seroprevalence of these viruses among humans and animals.

Rapid serological assays that do not rely on detection of neutralizing Ab with live virus requiring biocontainment are needed for detection of CVV-specific IgG and IgM. The MAC-ELISA format is extensively used in ADB’s diagnostic laboratory to detect IgM during an acute infection for several medically important arboviruses including DENV, WNV, St. Louis encephalitis virus, LACV, Eastern equine encephalitis virus, and CHIKV [49]. Since the MAC-ELISA specifically detects IgM, which is produced early in infection, acute diagnosis can be made in the absence of RT-PCR. Additionally, capture of IgM minimizes the incidence of false-negatives due to cross-reactive IgG that may be present in the sample if the patient has had prior exposure to a closely related virus.

Our results show the utility of these anti-CVV MAbs in a pilot MAC-ELISA. Four MAbs (CVV14, CVV15, CVV17, and CVV18) showed low nonspecific reactivity on NHS, while maintaining high reactivity with the CVV-positive human serum tested. MAb CVV14 exhibited the highest specificity when further evaluated in the pilot MAC-ELISA with eight human specimens. P/Ns for some of the samples were >3.0, the cutoff for a presumptive positive in the CDC MAC-ELISA format. This is most likely due to very low background from the use of purified virus in the assay resulting in lower OD readings of NHS on positive antigen. These lower OD readings most likely led to the false positive results in the assay. Additionally, the evaluation of the preliminary MAC-ELISA was limited by the small sample size. This was due to the limited number and volume of CVV positive human serum control available for use in the assay.

Currently, studies are underway for the development and optimization of a CVV MAC-ELISA for use in diagnostics utilizing MAb CVV14 as the detector Ab and evaluating different antigen preparations including tissue culture fluid and suckling mouse brain antigen. Likewise, development of human-chimeric IgM Abs utilizing variable regions from these anti-CVV murine hybridomas for use as positive controls in MAC-ELISA is in progress. Validation of this assay will be ongoing as more samples from recent CVV infections are identified for testing including specimens identified retroactively from patients exhibiting encephalitis with no known causal agent. The MAbs described here have shown to be cross-reactive with other members in the Bunyamwera serogroup and may be useful in future assays to detect related viral infections for which no rapid serological diagnostic assay exists.

Even though CVV circulates widely in mosquito and animal species and has a large geographic range, our understanding of the virus’s impact on human health is limited. Currently, we do not fully understand the spectrum of disease in humans caused by CVV including its potential as a teratogenic agent, which may be likely, given the strong correlation between maternal anti-CVV Abs and macrocephaly [21]. Only severe human cases have been identified to date without the utilization of rapid serological assays. Assays that do not rely on live virus will aid in making diagnostics more widely available in public health laboratories for CVV and other viruses in the Bunyamwera serogroup. Rapid and higher throughput serological assays utilizing these MAbs will make larger seroprevalence studies possible facilitating our understanding of the virus ecological life cycle, disease prevalence, and impact on at-risk populations.

## Author disclaimer

The findings and conclusions in this report are those of the authors and do not necessarily represent the official position of CDC.

## Supporting information

S1 Fig CVV-specific neutralizing antibody responses. Percent neutralization of serum samples taken from immunized mice 85-days PI (A) and purified MAbs (B) was calculated based on virus input titer. Virus neutralization curves were generated by a 4-parameter non-linear regression dose response and used to calculate the half-maximal inhibitory concentration (IC_50_) values of each sample using GraphPad Prism V6. The dotted line represents 50% neutralization.
(TIF)

S2 Fig Reactivity of anti-CVV MAbs with CVV, and control Abs with related orthobunyaviruses. Vero cells were grown on glass coverslips in 24-well plates, infected with orthobunyaviruses at an MOI 1.0 and fixed 24 hours post-infection. Cells were stained with Abs and a goat anti-mouse FITC labeled conjugate (green). Nuclei of cells were stained with DAPI (blue). A) MHIAF to several orthobunyaviruses was used as a positive control in IFA and tested on homologous virus-infected cells. A purified anti-alphavirus IgG2b antibody was used as a negative antibody control (NAC) to detect nonspecific binding in the assay. B) Reactivities of anti-CVV MAbs on CVV-infected cells and uninfected (UI) cells were included as controls in IFA experiments.
(TIF)

S1 Table Antibody isotypes determined using the Antibody Isotyping 7-Plex Mouse ProcartaPlex kit. (DOCX)
