# Supplementary figures and images for: Monoclonal antibodies to Cache Valley virus for serological diagnosis
Source: PLoS Negl Trop Dis. 2022 Jan 24;16(1):e0010156. doi: 10.1371/journal.pntd.0010156 (PMC8812937; doi:10.1371/journal.pntd.0010156)

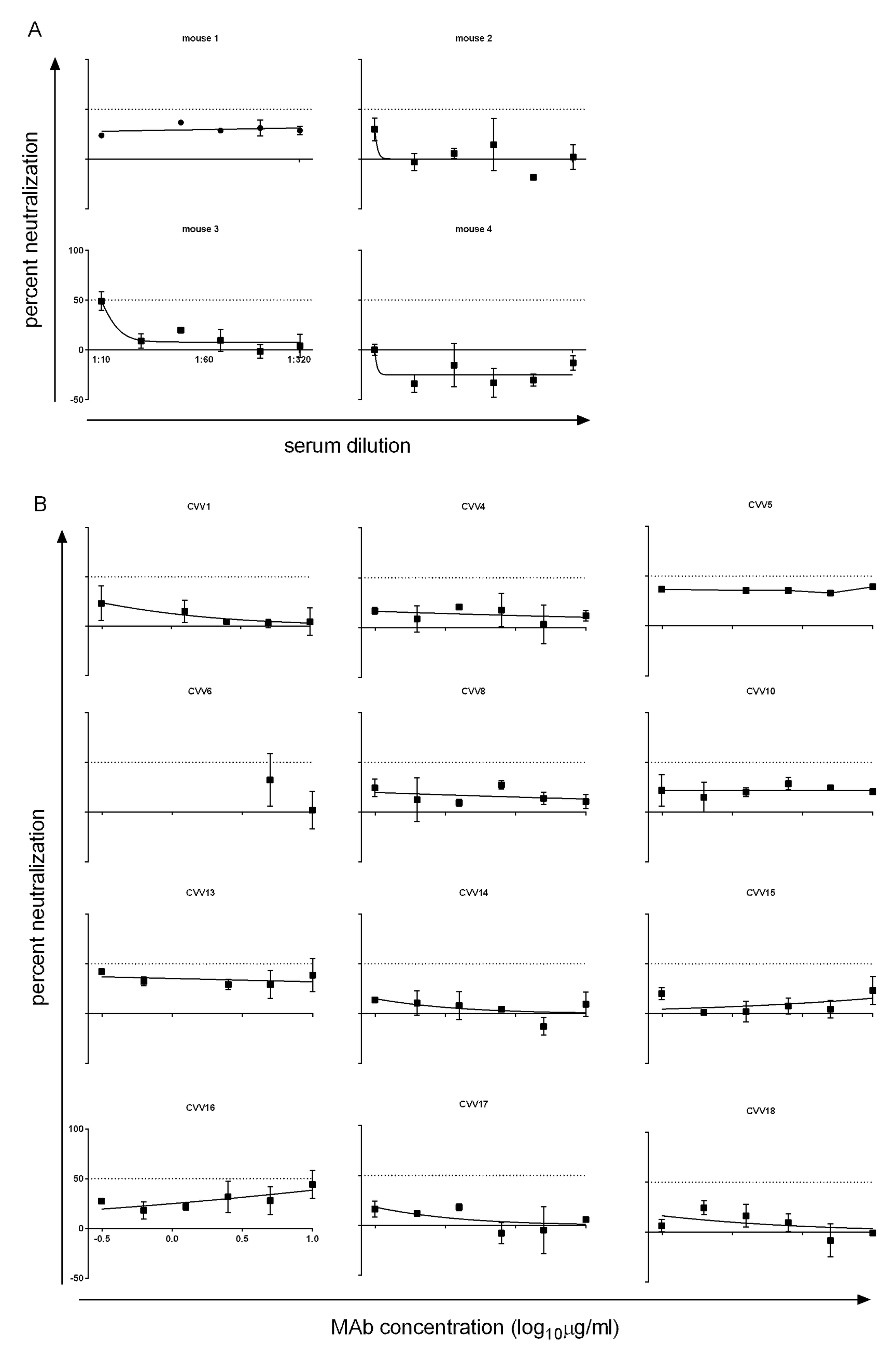

Supplement: S1 Fig — Percent neutralization of serum samples taken from immunized mice 85-days PI (A) and purified MAbs (B) was calculated based on virus input titer. Virus neutralization curves were generated by a 4-parameter non-linear regression dose response and used to calculate the half-maximal inhibitory concentration (IC50) values of each sample using GraphPad Prism V6. The dotted line represents 50% neutralization. (TIF) [file pntd.0010156.s001.tif]

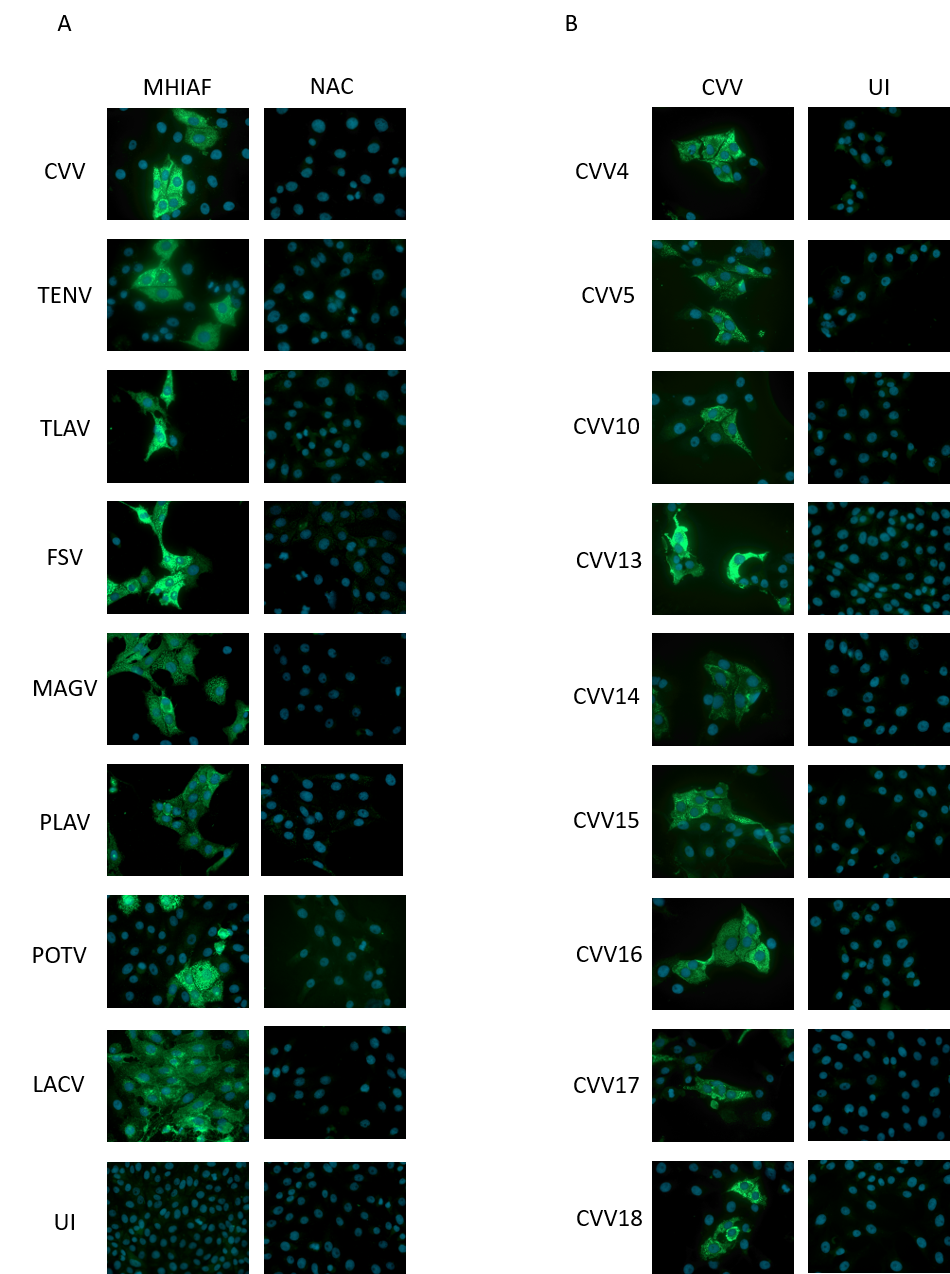

Supplement: S2 Fig — Vero cells were grown on glass coverslips in 24-well plates, infected with orthobunyaviruses at an MOI 1.0 and fixed 24 hours post-infection. Cells were stained with Abs and a goat anti-mouse FITC labeled conjugate (green). Nuclei of cells were stained with DAPI (blue). A) MHIAF to several orthobunyaviruses was used as a positive control in IFA and tested on homologous virus-infected cells. A purified anti-alphavirus IgG2b antibody was used as a negative antibody control (NAC) to detect nonspecific binding in the assay. B) Reactivities of anti-CVV MAbs on CVV-infected cells and uninfected (UI) cells were included as controls in IFA experiments. (TIF) [file pntd.0010156.s002.tif]
