# Supplementary material for: Monoclonal antibodies to Cache Valley virus for serological diagnosis
Source: PLoS Negl Trop Dis. 2022 Jan 24;16(1):e0010156. doi: 10.1371/journal.pntd.0010156 (PMC8812937; doi:10.1371/journal.pntd.0010156)
Supplement: S1 Table — (DOCX) [file pntd.0010156.s003.docx]

**S1 Table.** Antibody isotypes determined using the Antibody Isotyping 7-Plex Mouse ProcartaPlex kit.

| Sample *^a^* | Average Mean Fluorescent Intensity (±SD) | | | | | | |
| --- | --- | --- | --- | --- | --- | --- | --- |
|  | IgA | IgE | IgG1 | IgG2b | IgG2C | IgG3 | IgM |
| standard 1 | 1187 (49.49) | 1306.5 (74.24) | 7408 (126.6) | 6257.75 (248.5) | 4584.25 (225.2) | 996.5 (53.03) | 935.25 (3.181) |
| standard 2 | 1020.25 (1.06) | 768 (2.83) | 6556.5 (54.44) | 5379.25 (0.353) | 3818.5 (102.5) | 892.5 (36.06) | 596.25 (18.03) |
| standard 3 | 730 (16.97) | 339.5 (14.85) | 4633.75 (49.14) | 3314.25 (125.5) | 2538.25 (47.73) | 646 (2.828) | 298.75 (22.27) |
| standard 4 | 391.25 (7.425) | 127.5 (6.364) | 2253.5 (41.72) | 1442 (9.899) | 1162 (15.56) | 310 (5.657) | 122 (4.246) |
| standard 5 | 160.25 (7.425) | 43 (1.414) | 846.5 (14.14) | 478.25 (1.767) | 404.5 (13.43) | 109 (4.242) | 56.75 (3.181) |
| standard 6 | 63 (2.828) | 16 (1.414) | 292.75 (13.78) | 148.25 (15.91) | 126.5 (12.02) | 36.5 (2.828) | 33.5 (0.707) |
| standard 7 | 24.5 (2.121) | 9 (0.000) | 99.5 (0.707) | 60 (18.38) | 42.5 (2.121) | 13.5 (0.707) | 22.5 (2.121) |
| control 1 | 7 (0.000) | 5 (0.000) | 16 (1.414) | 5.75 (0.353)) | 5.5 (0.707) | 3.5 (0.707) | 16.5 (0.707) |
| CVV1 | 10 (0.000) | 11 (0.000) | 20 (0.000) | **6289.75 (486.8)** | 6 (0.000) | 12 (2.828) | 22 (1.414) |
| CVV4 | 11.5 (0.707) | 11 (0.000) | 20 (0.000) | **7690.25 (330.6)** | 6 (0.000) | 14 (0.000) | 23.5 (0.707) |
| CVV5 | 11 (0.000) | 12.5 (0.707) | 25.25 (0.353) | **8509 (161.2)** | 9.25 (0.353) | 15 (0.000) | 23 (1.414) |
| CVV6 | 7 (0.000) | 4.5 (0.707) | 18.5 (2.121) | **109 (90.51)** | 5.5 (0.707) | 4.25 (0.353) | 16.5 (0.707) |
| CVV8 | 8.5 (0.000) | 7.5 (0.707) | 18 (1.414) | **4654 (352.8)** | 5.5 (0.707) | 8.5 (0.707) | 19.5 (0.707) |
| CVV10 | 10 (0.000) | 10.5 (0.707) | 760.5 (119.5) | **6890.75 (774.6)** | 5.75 (0.353) | 16.5 (0.707) | 22.5 (0.707) |
| CVV13 | 8.5 (0.707) | 7.5 (0.707) | 18 (1.414) | **4623 (46.67)** | 6 (0.000) | 8.5 (0.707) | 19 (0.000) |
| CVV14 | 12 (0.000) | 12.75 (0.353) | 21.5 (0.707) | **7173 (343.6)** | 6.5 (0.707) | 15.5 (0.707) | 23 (0.000) |
| CVV15 | 13 (0.000) | 13.5 (0.707) | 20.25 (0.353) | **8994.75 (125.5)** | 6.5 (0.707) | 16.5 (0.707) | 23.5 (0.707) |
| CVV16 | 9.5 (0.707) | 9 (0.000) | 18.5 (0.707) | **6090.75 (309.4)** | 5.75 (0.353) | 11.5 (0.707) | 19.5 (0.707) |
| CVV17 | 6.75 (0.353) | 5 (0.000) | 16 (0.000) | **601.5 (60.10)** | 5 (0.000) | 4.5 (0.707) | 18 (1.414) |
| CVV18 | nd*^b^* | nd | nd | nd | nd | nd | nd |

*^a^*Average mean fluorescent intensity (±SD) was determined using supernatant from hybridomas secreting anti-CVV MAbs according to the manufacturer’s instructions. Seven standards and one negative control from the test kit were included in the assay.

*^b^*ND = not determined.

Values in bold indicate MAbs’ highest reactivity with murine isotype targets.
